# A Comparison of Biomarker Based Incidence Estimators

**DOI:** 10.1371/journal.pone.0007368

**Published:** 2009-10-07

**Authors:** Thomas A. McWalter, Alex Welte

**Affiliations:** 1 School of Computational and Applied Mathematics, University of the Witwatersrand, Johannesburg, South Africa; 2 DST/NRF Centre of Excellence in Epidemiological Modelling and Analysis (SACEMA), Stellenbosch University, Stellenbosch, South Africa; University of Cape Town, South Africa

## Abstract

**Background:**

Cross-sectional surveys utilizing biomarkers that test for recent infection provide a convenient and cost effective way to estimate HIV incidence. In particular, the BED assay has been developed for this purpose. Controversy surrounding the way in which false positive results from the biomarker should be handled has lead to a number of different estimators that account for imperfect specificity. We compare the estimators proposed by McDougal et al., Hargrove et al. and McWalter & Welte.

**Methodology/Principal Findings:**

The three estimators are analyzed and compared. An identity showing a relationship between the calibration parameters in the McDougal methodology is shown. When the three estimators are tested under a steady state epidemic, which includes individuals who fail to progress on the biomarker, only the McWalter/Welte method recovers an unbiased result.

**Conclusions/Significance:**

Our analysis shows that the McDougal estimator can be reduced to a formula that only requires calibration of a mean window period and a long-term specificity. This allows simpler calibration techniques to be used and shows that all three estimators can be expressed using the same set of parameters. The McWalter/Welte method is applicable under the least restrictive assumptions and is the least prone to bias of the methods reviewed.

## Introduction

Although prospective follow-up of an initially HIV-negative cohort is widely regarded as the “gold-standard” for estimating incidence, the idea of utilizing a biomarker to define a suitable class of “recently infected” individuals, and then to use the prevalence of this class as the basis for estimating HIV incidence, is attractive for a number of reasons. Since this can be implemented using a cross-sectional survey, it is logistically simpler, cheaper and less prone to the biases that result from intervention and loss to follow-up.

The BED capture enzyme immunoassay (BED assay) has been developed for this purpose [1], [2] and widely used [3]. It measures the proportion of IgG that is HIV-1 specific as a normalized optical density (ODn). Since this proportion increases over time after the infection event, specifying an ODn threshold allows seropositive individuals to be classified as recently infected, if they are below threshold, and as non-recently infected, if they are above threshold. Initially, an incidence formula was proposed [1] that did not explicitly account for the possibility of assay non-progressors (i.e. individuals who never develop enough of an immunological response to cross the threshold). This method was similar to the earlier approaches of Brookmeyer and Quinn [4], and Janssen *et al.* [5]. Later, the methodology proposed by McDougal *et al.* [6] was the first to deal with assay non-progressors. They derived an incidence formula which can be expressed in terms of the prevalence of below-threshold seropositive, above-threshold seropositive and seronegative individuals, and four assay calibration parameters, being the mean window period (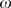), sensitivity (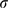), short-term specificity (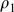) and long-term specificity (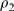). Introducing the long-term specificity parameter provided a way to quantify assay non-progression.

Two other incidence paradigms that explicitly account for assay non-progressors have since been formulated. Hargrove *et al.* [7] proposed a simpler incidence estimator which is equivalent to the McDougal estimator when one sets 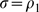. Recently, we have also proposed a formally rigorous incidence paradigm [8], which accounts for assay non-progression using fewer assumptions than are made by McDougal *et al.* The parameters that emerge naturally in our estimator are a mean window period and a probability of not progressing on the assay (which can also be expressed as a long-term specificity).

A large portion of this paper is dedicated to an analysis of the assay parameters of the McDougal methodology, showing how they are related. By using a survival analysis formulation of the problem, we are able to write down precise expressions for the parameters. This allows us to derive a relationship between three of the parameters, which simplifies the McDougal estimator by showing that only 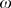 and 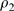, which are considerably easier to calibrate than 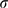 and 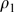, are required in the final formula. The reduction of the McDougal approach is important in that it shows that all three incidence estimators are, in effect, based on the same underlying parameters characterising the performance of the assay, and are therefore amenable to direct comparison.

We then compare the performance of the three incidence estimators by substituting analytic expressions for population counts, derived from a model steady state epidemic, into the various formulae. This analysis shows that only our formula [8] produces a bias-free result. Although the biases are typically small, we demonstrate, using numerical examples, that there are regimes where bias may be significant.

The paper is structured as follows: We start by describing the McDougal methodology and, in doing so, write down mathematical expressions for the assay calibration parameters. In the next section we restate the assumptions made by McDougal *et al.* in a mathematically precise manner. This allows us to derive the identity that shows the relationship between the parameters. We then present the three incidence formulae and compare them by inserting the population counts from a model steady state epidemic. We conclude the paper with a discussion of the implications of the identity and the steady state analysis.

## Analysis

### The McDougal Methodology

Denote the number of individuals in a cross-sectional sample who are respectively *under*-threshold, *over*-threshold and *healthy* (susceptible) by 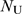, 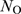 and 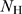. Then the McDougal estimator [6] can be written as
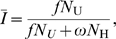
(1)where 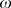 is specified in years and the “correction factor”,
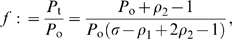
(2)is the ratio of the “true” proportion 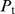 of recent infections and the proportion 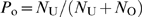 of the HIV positive individuals that are under the threshold. This correction factor, which depends on subtle definitions for the sensitivity and specificity parameters, explicitly accounts for the fact that the BED assay imperfectly classifies individuals as “recently infected”.

McDougal *et al.* calibrate these parameters using seroconversion panels which show BED optical density as a function of time since infection (some of these are published [1], [2]). The calibration occurs in two stages. A window period is estimated, and then estimates of the sensitivity, short-term specificity and long-term specificity are determined with respect to the window period.

The window period is estimated as “the mean period of time from initial seroconversion to reaching an ODn of 0.8” [6]. Although it is not explicitly stated, we presume that those individuals that never reach the threshold, either because they do not progress above the threshold or because they die before reaching the threshold, are not included in the calculation of the mean. More specifically this implies that the window period is the mean observable threshold crossing time, conditional on assay progression (i.e. actually reaching the threshold).

In order to calibrate the sensitivity, short-term specificity and long-term specificity, “a plot of the proportion of specimens positive in the assay versus time since seroconversion” is generated (also later referred to as “the curve”). This is the sampled survival function (essentially a Kaplan-Meier curve) in the state of being *under* the threshold, conditional on being *alive*, which we denote 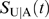.

The sensitivity of the test is estimated for an interval corresponding to the window period by “integrating the curve within the window”. Short-term specificity is calculated for “the interval immediately after, and equal in duration to, the window period”. Long-term specificity is for “the period thereafter (where the curve is flat)”. McDougal *et al.* explicitly make the following assumptions, with the justification that they “are reasonable as very little attrition (from death) during the first two time intervals after infection would be expected”:

1. “Recent infections are randomly distributed within the first window period”.
2. “The number of persons in the interval of equal duration immediately after the mean window period equals the number in the first window period”.
3. “The remainder of the population is more than two window periods since seroconversion”.

**Figure 1:**
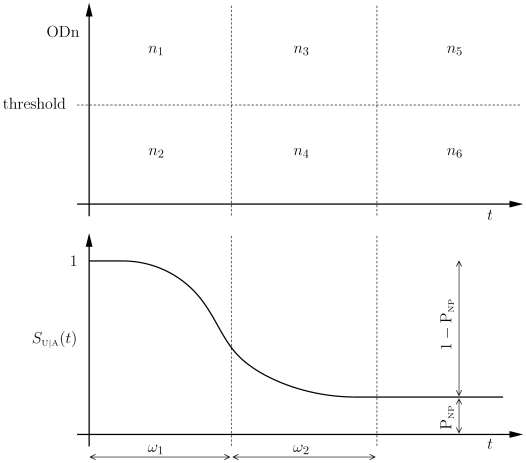
The six sector model of McDougal et al. The top graph shows counts 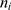 and the bottom graph shows the survival function 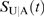 versus time since infection.

While it may be true in the situation being explored here, we note that it is not *a priori* obvious that the choice of equal window periods ensures that 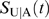 is flat after twice the window period. With this in mind, we propose a generalization in which there are two window periods with arbitrary values 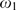 and 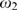, chosen so that all individuals that progress do so in a time less than 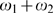 after seroconversion (i.e. 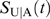 is flat for 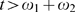, see the bottom graph of Figure 1). It should be noted that this is a special survival curve in that it never reaches a zero value, capturing the fact that a certain proportion of individuals will never progress above the threshold. This is what differentiates this approach from other approaches that do not account for assay non-progression (Such as Brookmeyer and Quinn [4], Janssen *et al.* [5], and Parekh *et al.* [1]).

For analytical convenience, we introduce 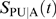, the survival of assay *progressors* in the state of being *under*-threshold. We also introduce 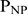, the probability of individuals *not progressing* on the assay. Then 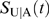, 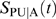 and 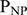 are related by
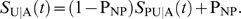



The introduction of 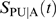 allows us to provide a precise definition of the window period used by McDougal *et al.* It is the mean time between seroconversion and reaching threshold, for individuals who progress:
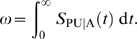
(3)


Assumption 1 above can only mean that infection times in the first window period are *uniformly distributed*. Although assumption 2 merely states that the number of infections in the second window period is equal to the number in the first, we shall see later that for 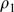 to be a property of the assay, independent of the epidemic state, we require the stronger assumption that the infection events in the second window period are also *uniformly distributed* with the same intensity as in the first window period. We see below that this assumption is implicit in the work of McDougal *et al.* To make this more explicit, we define 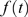 to be the density of times since infection realized in the sample. The number of *seropositive* individuals is then given by
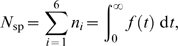
where 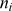 are the counts of individuals in the various categories depicted in the top graph in Figure 1.

Setting 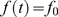 over the first two window periods means that the ratio of the number of infected individuals in the second window period to those in the first period is 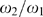. Assumption 2 is recovered when the length of the window periods is equal. It should be noted that 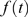 depends on incidence, susceptible population and life expectancies over the history of the epidemic. With reference to Figure 1, we are now in a position to write expressions for the number of seropositive individuals in each sector:
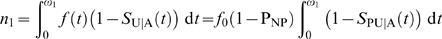


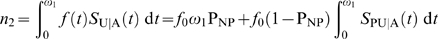


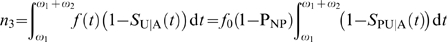


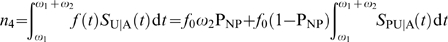


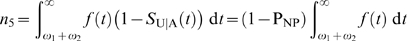


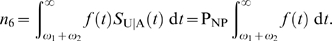



Using the above expressions, the sensitivity, the short-term specificity and the long-term specificity are given by
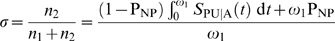


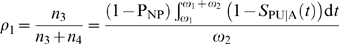


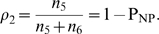



We can now see why the assumption of uniformly distributed infection events for the first and second window periods is required – it is the only way in which a cancelation of 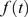 in the expressions for 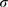 and 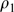 is possible. Note that under bias-free recruitment into a survey, at time 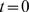, we have
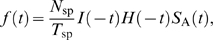
(4)where 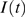 is the instantaneous incidence, 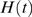 is the number of healthy (susceptible) individuals, 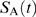 is the life-expectancy survival function measured from the time since infection and
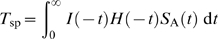
is the total number of seropositive individuals alive in the population at the time of the survey. The ratio 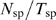 is just the fraction of the total population that has been recruited. Thus, the only sensible way to ensure that 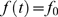 for 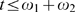, is to assume that the incidence and the susceptible population are constant, and the survival function 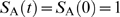.

We also see why 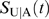 must be flat after both window periods – this ensures that 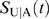 is constant and can be pulled out of the integrals in the expressions for 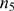 and 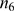 as the factor 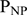. This is necessary for 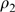 to be independent of 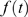.

Furthermore, in order to specify 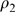 so that it is independent of the state of the epidemic, an implicit assumption is being made that survival is the same for assay progressors and assay non-progressors. Note that 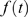 appears in the expressions for both 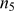 and 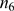. If different life expectancies were used in these formulae, reflecting a difference in survival for assay progressors and assay non-progressors, the 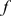
′s in these formulae would need to be different, and would not cancel in the expression for 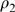. This assumption is not explicitly stated by McDougal *et al.* but is implicit in their assumption that 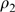 is independent of epidemic state.

With the calibration parameters specified in the more general setting of unequal window periods 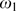 and 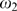, we now generalize the expression for the correction factor
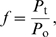
where 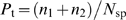 is the proportion of seropositive individuals who are *truly* infected at a time less than 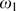. Recalling that 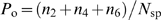 and using the definitions of the parameters, it is easy to verify that
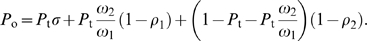



This means that the correction factor can be expressed as
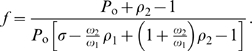
(5)


Note that this equation simplifies to the previous expression (2) when one sets 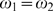.

### Elimination of Parameters

For completeness, we now provide a precise specification of the assumptions that are required in order to facilitate the analysis in the rest of this paper. We note that with the exception of arbitrary sized window periods, these assumptions are equivalent to the assumptions – either explicit or implicit – that are being made by McDougal *et al.* [6].

#### Model Assumptions

*Specify window periods* 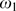 *and* 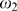 *. We assume that:*

1. *The window periods are chosen so that the survival function* 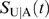 *is flat (and equal to* 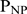 *) for* 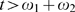 *. This means that* 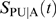 *only has support on the time interval* 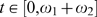 *.*
2. *Arrival times of infection events are uniformly distributed on the interval* 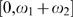 *. An equivalent way of stating this assumption is that over the interval* 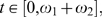 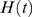 *and* 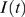 *are constant and* 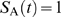 *.*
3. *Survival is the same for assay progressors and assay non-progressors.*

We are now able to provide the identity relating the parameters in the McDougal approach.

#### Proposition 1

*Under the model assumptions stated above, the following identity holds:*

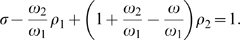
(6)


#### Proof

Since we assume that 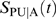 only has support on 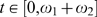, we have
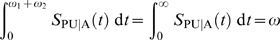



Then, simply evaluating
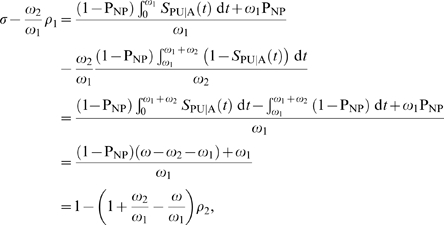
yields the result directly.

Using the proposition, the correction factor (5) simplifies to
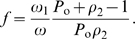



This expression no longer relies on estimates for 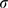 and 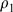. It is also interesting to note that it does not depend explicitly on 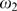. Calibrating 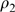, however, requires identifying individuals who have been infected for *at least* 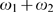. Thus, 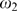 need not be precisely known, but a safe upper bound for 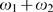 is required.

Furthermore, if we set 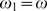 as in McDougal *et al.* then we recover
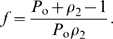
(7)


Note that (2) as stated in McDougal *et al.* contains three calibration parameters (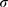, 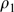 and 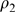), while (7) contains only one calibration parameter (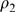). Incidence estimates using (1) and (7), however, still require the estimation of 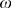. The method of McDougal *et al.* can in principle be applied to an arbitrarily declared (as opposed to measured) window period, as long as 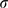, 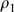 and 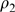 are calibrated for that value. We have therefore reduced the number of calibration parameters by one.

Estimation of extra parameters may unnecessarily dilute the statistical power of the calibration data at hand. Moreover, estimates of the uncertainty due to calibration, based on the assumption of the independence of 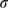, 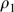 and 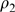, will be incorrect. Note that when one sets 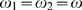, the identity is reduced to
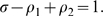



Substituting the estimates of the parameters found by McDougal *et al.*, namely 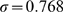, 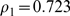 and 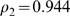, into this equation gives a value of 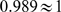 for the left hand side. The slight discrepancy is a manifestation of the combined fluctuations in the estimates of 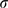, 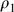, 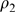 and 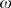. Although 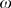 is superficially absent in the identity, it enters as the period over which the other parameters are defined.

When one assumes that 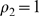 (corresponding to the situation where there are no assay non-progressors) and 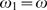, the identity reduces to
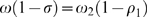
(8)and the ratio of counts over this period is given by
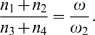



Using this ratio and substituting the definitions for 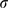 and 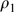 into (8) yields 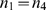. Therefore, for tests with perfect long-term specificity, the observed count of individuals who are under-threshold is an unbiased estimate of the number of infections in the last period 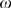. This was noted in a less general analysis of Brookmeyer [9] where assay non-progressors were *a priori* excluded.

It should be noted that there is a subtlety in the definition of the window period that emerges in the above analysis. If, instead of (3), the window period is defined by
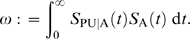
(9)then the two definitions are equivalent under the model assumptions leading to the proposition. This follows from the fact that 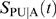 only has support on 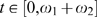 and that 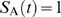 over that interval. We have suggested an alternative incidence estimation paradigm [8] which requires fewer assumptions than the method of McDougal *et al.* In this approach 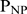 and 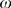, as defined in (9), emerge as the natural calibration parameters.

### Comparison of Estimators Under Steady State Conditions

We now provide a simplified form for the McDougal incidence estimator based on the proposition. Substituting the new correction factor (7) into their estimator (1) and expressing the result in terms 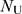, 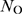 and 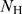 gives
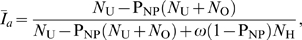
(10)where 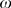 is specified in years. Here the subscript 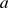 indicates that the estimator is quoted as an “annualized incidence”. Note that in writing down this expression, we have chosen to use 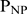 rather than the long-term specificity as this is a biologically more intuitive parameter. In addition, the other two estimators to which this estimator will be compared were originally specified in terms of 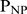.

In a previous attempt to simplify the McDougal formula, Hargrove *et al.* [7] proposed the following incidence formula
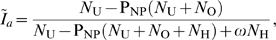
(11)where 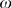 is specified in years. Note that they use the symbol 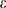 where we use 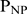.

We have recently rigorously derived a weighted incidence estimator under less restrictive assumptions than those that are required for the McDougal or Hargrove approach [8]. Unlike the other two estimators, our estimator is expressed as a rate (indicated by a subscript 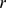) and is given by
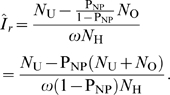
(12)


To convert between an annualized incidence and an incidence expressed as a rate, one can use the standard conversion formula
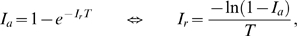
where 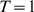 year.

In Appendix S1 we show that, under steady state conditions, 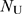
and 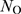 are specified in terms of 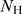 and an incidence rate 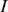 as
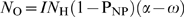
(13)and
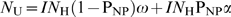
(14)where 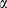 is the post-infection life expectancy. Using these population counts, it is now possible to compare the performance of the incidence estimators. Substituting (13) and (14) into the McDougal formula (10) yields
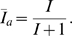



Converting this to a rate, we have
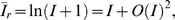
where the last step results from a Taylor series expansion. Thus the estimator is accurate for small values of 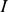, but yields a discrepancy at 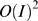. The reason for this discrepancy is subtle. In deriving the correction factor, McDougal *et al.* assume uniform infection events over the window periods. We have shown that this is consistent with assuming that the incidence and susceptible population are constant. In using this factor to estimate an incidence with (1) they have, however, inconsistently assumed that these infection events are generated in a susceptible population which is being depleted by the infection events over a period of a year. This is implied by their choice of denominator in that formula, which adds back an annualized number of recent infections into the susceptible population. This is at odds with the assumption of a constant susceptible population, and leads to dimensionally inconsistent incidence estimators, (1) and (10).

**Figure 2:**
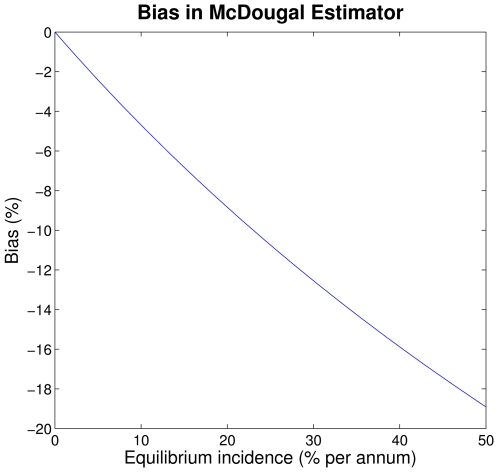
Bias in the McDougal estimator. Relative difference between the McDougal estimate and the equilibrium incidence plotted as a function of equilibrium incidence.

To illustrate the magnitude of the bias, Figure 2 shows the difference between the McDougal incidence estimate and the equilibrium incidence, expressed as a percentage. Note that the range of incidence values used is large (up to 50% per annum). Although incidence for HIV is not likely to be larger than about 15% in the highest risk groups (e.g. injection drug users [10]), if this methodology were used to monitor other rapidly spreading epidemics, where incidence is large when stated in units of years, it would certainly produce unacceptable bias.

Substituting the counts into the Hargrove formula (11) yields
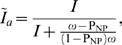
which, when converted to a rate, gives
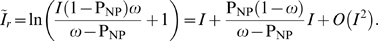



**Figure 3:**
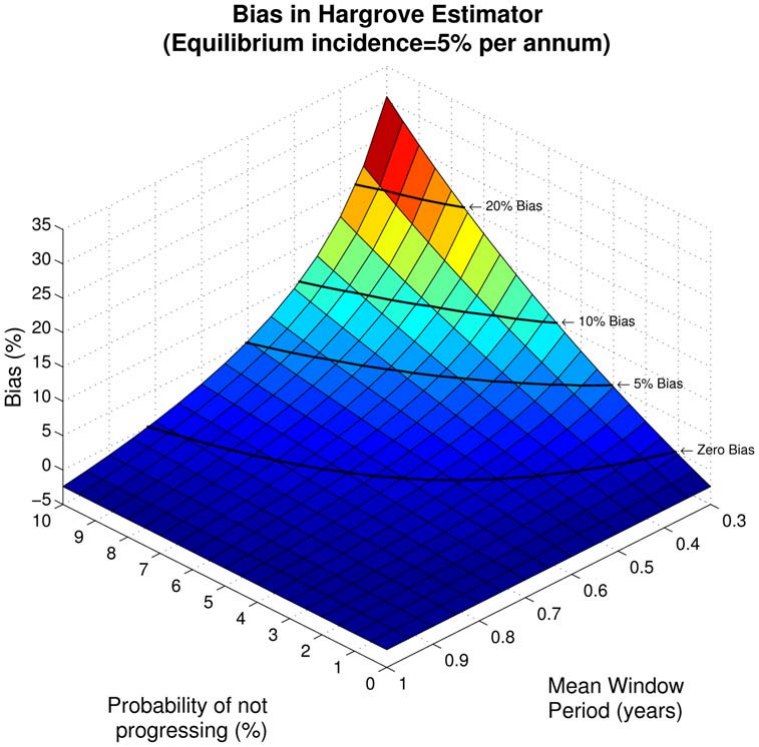
Bias in the Hargrove estimator. Relative difference between the Hargrove estimate and the equilibrium incidence plotted as a function of 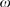 and 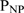 for an equilibrium incidence of 5% per annum. Black lines indicate contours of equal bias.

The Hargrove estimator incorporates the same form of denominator which leads to the second order discrepancy and dimensional inconsistency in the McDougal formula, and, in addition, it includes a linear bias term. Figure 3 demonstrates the bias introduced as a function of 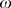 and 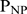 for an equilibrium incidence of 5% per annum. Although the bias is worst in the regimes where all the estimators have little statistical power and are unlikely to be used, there are nevertheless intermediate regimes where the bias is significant. Note that the estimator produces the same result (and bias) as the McDougal estimator when 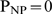 or 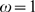.

Finally, substituting the counts into our formula (12), which is already specified as a rate, yields
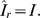



Thus, under the assumption of a steady state epidemic, our weighted incidence estimator recovers the steady state incidence exactly. It is also the maximum likelihood estimator. This can be seen by writing the estimator in terms of the population proportions
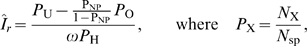
and noting that, since the counts are trinomially distributed, the sample proportions are the maximum likelihood estimates of the population proportions. We have already seen that the estimator solves for the equilibrium incidence. Thus, by the invariance property of maximum likelihood estimators (see e.g. p. 105 of van den Bos [11]), it is the maximum likelihood estimator for the incidence. This has also recently been demonstrated by Wang and Lagakos [12] by explicit maximization of the log likelihood function.

A weighted incidence will in general not be equal to the instantaneous incidence under non-steady state conditions. We should, however, demand that any incidence formula exactly recover the incidence under this rather idealized situation.

## Discussion

We have shown that under a precise restatement of the McDougal *et al.* assumptions, there exists a redundancy in the parameters they chose to characterise the assay. This allows the elimination of 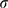 and 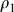 from their estimator, with the important advantage that the remaining parameters are easier to calibrate. The calibration of 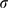 and 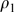 requires obtaining specimens from individuals with confidence about their time since infection (i.e. using frequent follow-up). On the other hand both 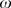 and 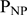 (or equivalently 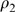) can be estimated through long follow-up intervals. The estimate for 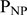 is the proportion of under-threshold samples known to be obtained more than 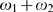 post-infection. Given an estimate for 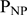, an estimate of 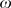 can be obtained from data with follow-up intervals greater than 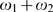 using an extended version [13] of the Bayesian approach previously described by Welte [14].

We have also shown that under steady state conditions the only estimator that is dimensionally consistent and produces an unbiased result is the one we have previously derived [8]. It is also the maximum likelihood estimator. The new approach makes fewer assumptions than the other methods. In particular, it consistently accounts for a dynamic epidemic by adopting a weighted definition of incidence. This overcomes a drawback of the other two methods which assume epidemic equilibrium for at least a period equal to the maximum progression time (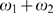). It should be noted that this methodology is applicable to any biomarker, not only the BED assay – all that is needed is a suitable calibration of the assay parameters. It also follows that cross-sectional incidence estimates using this approach are applicable to infections other than HIV, as long as suitably calibrated assays that test for recent infection are available.

A shortcoming of all the methods explored here is that they make the assumption, either implicitly or explicitly, that survival for assay non-progressors and assay progressors is the same. As we have shown, relaxing this assumption means that the long-term specificity becomes epidemic state dependent and hence is time dependent. We are involved in ongoing work to address this issue.

## Supporting Information

Appendix S1 (0.04 MB DOC)
