# Supplementary material for: A Comparison of Biomarker Based Incidence Estimators
Source: PLoS One. 2009 Oct 7;4(10):e7368. doi: 10.1371/journal.pone.0007368 (PMC2753643; doi:10.1371/journal.pone.0007368)
Supplement: Appendix S1 — (0.04 MB DOC) [file pone.0007368.s001.doc]

**Appendix S1**

We derive the population level counts associated with a steady state epidemic. Assume that the number of susceptible individuals and the incidence are constant, and that the sample for our incidence calculation consists of the entire population. Let the susceptible population be $H(t)=N_{\text{H}}$ and the incidence, expressed as a rate, be $I(t)=I$. Since our sample is the whole population we have $N_{\text{sp}}=T_{\text{sp}}$. Then, from (4) we obtain

$f(t)=IN_{\text{H}}S_{\text{A}}(t)$,

and the number of over-threshold and under-threshold individuals in the total population are given by

$$N_{\text{O}}=n_{1}+n_{2}+n_{3}$$

$$=\int_{0}^{\infty} f(t)(1-S_{\text{U|A}}(t))\text{ d}t$$

$$=IN_{\text{H}}(1-P_{\text{NP}})\int_{0}^{\infty} S_{\text{A}}(t)-S_{\text{PU|A}}(t)S_{\text{A}}(t)\text{ d}t$$

$$=IN_{\text{H}}(1-P_{\text{NP}})(\alpha-\omega)$$

and

$$N_{\text{U}}=n_{2}+n_{4}+n_{6}$$

$$=\int_{0}^{\infty} f(t)S_{\text{U|A}}(t)\text{ d}t$$

$$=IN_{\text{H}}\int_{0}^{\infty} (1-P_{\text{NP}})S_{\text{PU|A}}(t)S_{\text{A}}(t)+P_{\text{NP}}S_{\text{A}}(t)\text{ d}t$$

$=IN_{\text{H}}(1-P_{\text{NP}})\omega+IN_{\text{H}}P_{\text{NP}}\alpha$,

where $\alpha$ is the post infection life expectancy. It must be stressed that the survival functions $S_{\text{A}}(t)$ and $S_{\text{PU|A}}(t)$ are arbitrary. Thus, apart from assuming constant incidence and susceptible population, this is a quite general model.
